# Uncertainty in estuarine extreme water level predictions due to surge-tide interaction

**DOI:** 10.1371/journal.pone.0206200

**Published:** 2018-10-26

**Authors:** Charlotte Lyddon, Jenny M. Brown, Nicoletta Leonardi, Andrew J. Plater

**Affiliations:** 1 Department of Geography and Planning, University of Liverpool, Liverpool, United Kingdom; 2 National Oceanography Centre Liverpool, Joseph Proudman Building, Liverpool, Merseyside, United Kingdom; Centro de Investigacion Cientifica y de Educacion Superior de Ensenada Division de Fisica Aplicada, MEXICO

## Abstract

Storm surge is often the greatest threat to life and critical infrastructures during hurricanes and violent storms. Millions of people living in low-lying coastal zones and critical infrastructure within this zone rely on accurate storm surge forecast for disaster prevention and flood hazard mitigation. However, variability in residual sea level up-estuary, defined here as observed sea level minus predicted tide, can enhance total water levels; variability in the surge thus needs to be captured accurately to reduce uncertainty in site specific hazard assessment. Delft3D-FLOW is used to investigate surge variability, and the influence of storm surge timing on barotropic tide-surge propagation in a tide-dominant estuary using the Severn Estuary, south-west England, as an example. Model results show maximum surge elevation increases exponentially up-estuary and, for a range of surge timings consistently occurs on the flood tide. In the Severn Estuary, over a distance of 40 km from the most upstream tide gauge at Oldbury, the maximum surge elevation increases by 255%. Up-estuary locations experience short duration, high magnitude surge elevations and greater variability due to shallow-water effects and channel convergence. The results show that surge predictions from forecasting systems at tide gauge locations could under-predict the magnitude and duration of surge contribution to up-estuary water levels. Due to the large tidal range and dynamic nature of hyper-tidal estuaries, local forecasting systems should consider changes in surge elevation and shape with distance up-estuary from nearby tide gauge sites to minimize uncertainties in flood hazard assessment.

## 1. Introduction

Storm surges occur in coastal zones worldwide and correspond to short-term variations in sea-level driven by winds and atmospheric pressure changes associated with storms, tropical hurricanes and typhoons [1]. However, the concurrence of storm surges and astronomical tides presents a combined flood hazard, elevating observed water levels above the predicted tide, to create an extreme water level at observed high water [2,3]. The interaction between tide and surge can alleviate flood hazard when the storm surge peak does not coincide with high tide water, as may occur in some coastal and estuarine areas during storms [4]. Evidence of the damage caused by this combined flood hazard to coastal communities and critical infrastructure is well documented for the UK [5–7] and worldwide [8]. Severe storm surge events, such as the 1953 North Sea storm surge [5] and the 28 foot surge generated by Hurricane Katrina in Mississippi in 2005 [8], present a serious threat to coastal communities, with an increased risk of loss of life and damage to property. Severe flood events in recent decades and increasing numbers of assets within the coastal zone [9] have led to an increased prevalence of risk-based coastal planning frameworks [10].

Risk-based coastal planning frameworks rely on accurate water level boundary conditions, i.e. input data, to drive model simulations of flood events, which are representative of probabilistic extreme water levels within impact model assessments [11]. However the accurate representation of total water levels, which form the basis of flood hazard assessments in heavily populated and industrialized coastal zones, can be undermined by the variability in the combined forcing of extreme water levels. This variability can lead to uncertainty in flood hazard assessments, which poses a problem to policy makers and coastal planners as this uncertainty can result in underestimation of the severity and implications of extreme water levels [12].

Variability in the combined effect of tide-surge propagation is of particular significance in hyper-tidal estuaries, where the tidal range exceeds 6 m due to bathymetry of the estuary funneling and amplifying tidal wave propagation [13]. Even small changes in the magnitude or timing of a surge will contribute to increased total water levels and can be catastrophic if happening during high tide [4,14]. Catastrophic flooding experienced in the Bay of Fundy, Canada during the Groundhog Day Storm of 1976 would have been lessened if the peak of the surge arrived 1 hour before or after tidal high water [15]. The combined forcing of extreme water levels, notably tide and storm surges, can interact with each other in shallow water regions to alter the phase and amplitude of tidal high water [5], as shown by examples in the English Channel [16], Taiwan coast [17], and Queensland coast [18]. Interaction effects are largely a function of storm surge magnitude and can vary spatially across hyper-tidal estuaries, as shown in the Bay of Fundy where interaction effects are most strongly felt in the Northumberland Strait [19]. Storm surges can also enhance tidal wave propagation in estuaries and shallow coastal waters [20], as in the Bay of Bengal where advancement of high water can result in increased flood hazard [21]. Due to the importance of surge magnitude and timing relative to tidal high water in a hyper-tidal estuary [14], a precise surge prediction is required, in combination with the predicted tide, for estimation of total water levels for flood hazard assessment [22]. Current methods for storm surge prediction are limited in their accuracy as they may consider tide and surge as independent processes [23], and rarely consider the importance of a coupled tide-surge interaction component or physical processes e.g. funneling or seiches [24]. This paper will show there is a need to understand the variability of combined, coupled tide-surge boundary conditions to enable accurate representation of total water levels (prescribed here as a mean sea level, astronomical tidal curve, representative surge curve and freshwater input) for warning and flood hazard mitigation [25–27].

Hydrodynamic, numerical models can be used to assess the variability of coupled physical processes controlling estuarine water levels to minimize uncertainty in forecasting systems for flood hazard assessments [9,28]. Hydrodynamic models, which solve the shallow water, Navier-Stokes equations, are often used as a tool to simulate extreme water levels and assess uncertainty of storm surge elevation due to tide-surge interaction [19], bottom friction [29], land cover [30] and wave setup [31]. Sensitivity analyses allow input parameters to be varied one factor at a time to help distinguish which sources of uncertainty have most impact on an output total water level [32].

Uncertainty related to storm surge water level has been investigated by varying elevation of the storm surge, duration of the storm surge and timing of the peak of the storm surge with respect to the peak of the normal high tide [33]. The Environment Agency in the UK advises phase shifts in the timing of design surge curves relative to tidal high water for shoreline management planning [34]. This methodology captures the full range of potential outcomes of an extreme water level event throughout a model simulation. Detailed analysis of a residual surge improves understanding of tide-surge propagation and identifies where variability in surge elevation occurs through the tidal cycle.

Uncertainty can also be accounted for in an operational context [2]. For flood forecasting purposes, an ensemble of predicted storm surge conditions are combined with the predicted tide to determine the range of likely high water level that will be observed (a parameter known as ‘skew surge’) [35]. ‘Skew surge’, i.e. predicted astronomical high tide–nearest observed high tide, is a key indicator for flood hazard to evaluate absolute water level and understand error or sensitivity to surge timing and estuary morphology [35]. For flood management planning purposes, design surge curves are used to scale tidal simulations such that an extreme water level representing a required storm severity is generated (e.g., a 0.5% annual probability event [12]). In some studies uncertainty within the shape of the total water level curve is also considered [10] as this also impacts the duration of flooding or flood hazard at a defense. The assessment of coastal resilience to flooding along managed coastlines requires a good understanding of the site-specific flood hazard. Understanding of the uncertainty surrounding potential hazard from extreme water level forecasts issued at nearby locations is critical for monitoring defense performance and making informed decisions surrounding the delivery of shoreline management strategies over planning epochs (typically 0–20, 20–50 and 50–100 years for shoreline management in the UK [36]).Variability associated with storm surges in estuaries can be analyzed to minimize uncertainty in forecasting systems and storm impact assessments.

This research simulates the tide-surge propagation in a complex, coastal region, to assess the sensitivity of the surge, including a tide-surge interaction component, to storm timing relative to tidal high water, using the Severn Estuary, south-west England as an example of a hyper-tidal estuary. For the purposes of this paper the “Severn Estuary” is taken to include the Bristol Channel. This research uses the Severn Estuary as test case as it represents one of the most extreme examples worldwide in terms of tidal range and flood occurrence severity [37]. The Severn Estuary region exhibits the second largest mean spring tidal range in the world which increases from 6.2 m in the outer Bristol Channel to 12.20 m at Portbury [38]. Approximately 120 km^2^ of the Somerest Levels are at or below sea-level, and these floodplains historically suffer regular inundation [39]. The large tidal range and frequency of storm surges can increase flood hazard on heavily populated and industrialized, low-lying floodplains. The paper aims to assess changes in the storm surge at 5 tide gauge locations along the coast of the Severn Estuary (Hinkley Point, Newport, Portbury, Oldbury and Sharpness) and through the thalweg of the estuary. The modeled surge residual, herein termed the surge, contains a meteorological component and a tide-surge interaction component, and is isolated from the total water level by removing the modeled tidal signal [40]. The results (Section 3) show there is a need to capture uncertainties associated with storm surge elevation and shape in representative surge curves for flood risk assessments or forecast surge residuals when applied up-estuary of the tide gauge at which they are generated. Therefore the methodology and results could be applied to other hyper- and macro-tidal estuaries worldwide, but testing this is beyond the scope of the present study.

## 2. Methods

### 2.1 Delft3D and model domain

**Fig 1:**
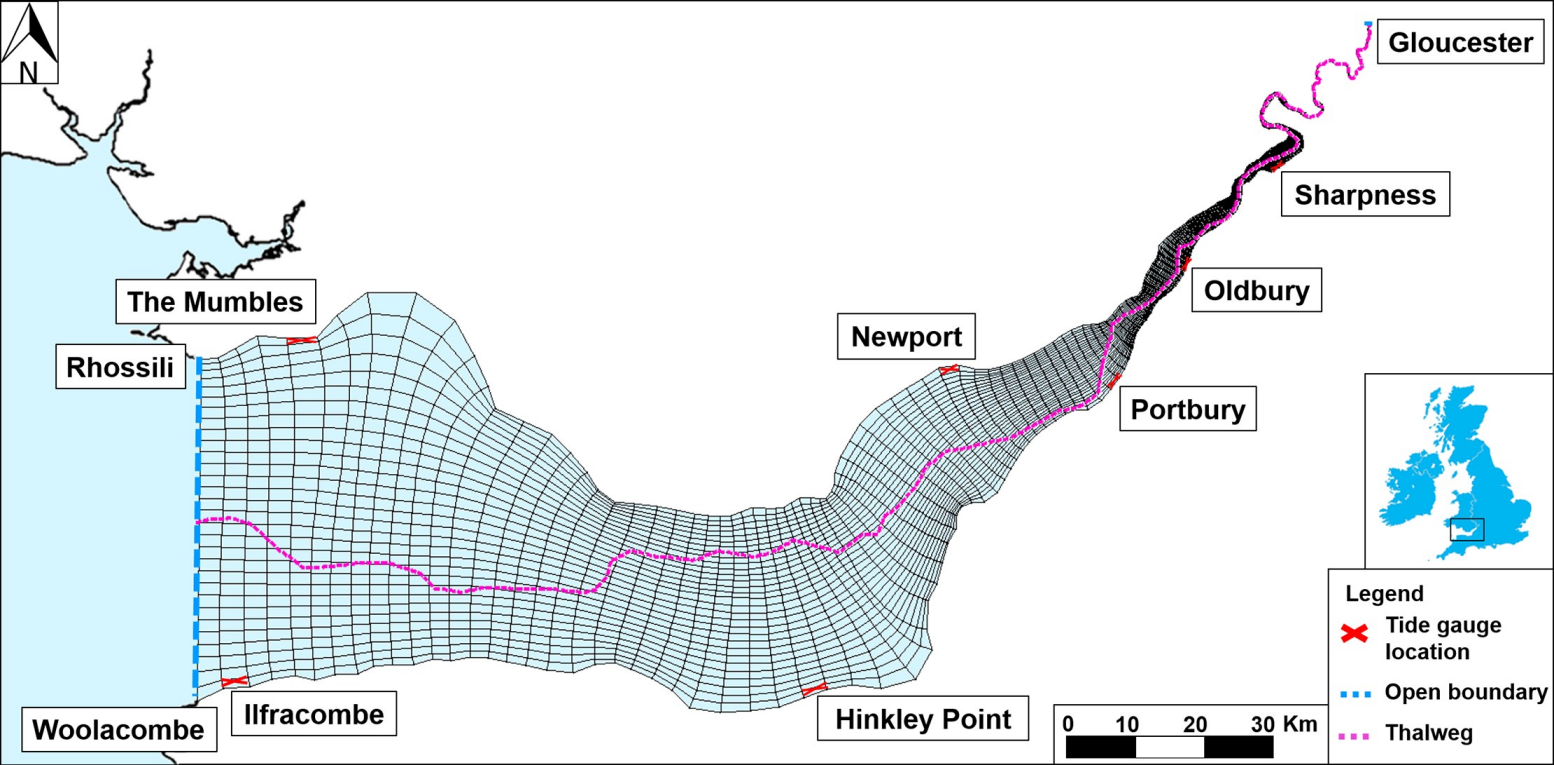
Severn Estuary model domain extending from Ilfracombe (51°12.668'N, 4°6.743'W) and the Mumbles (51°34.203'N, 3°58.534'W) in the west, to Gloucester (52° 89.3020’N, -2°2. 6361’W) in the east. The bathymetry is relative to chart datum (CD).

**Fig 2:**
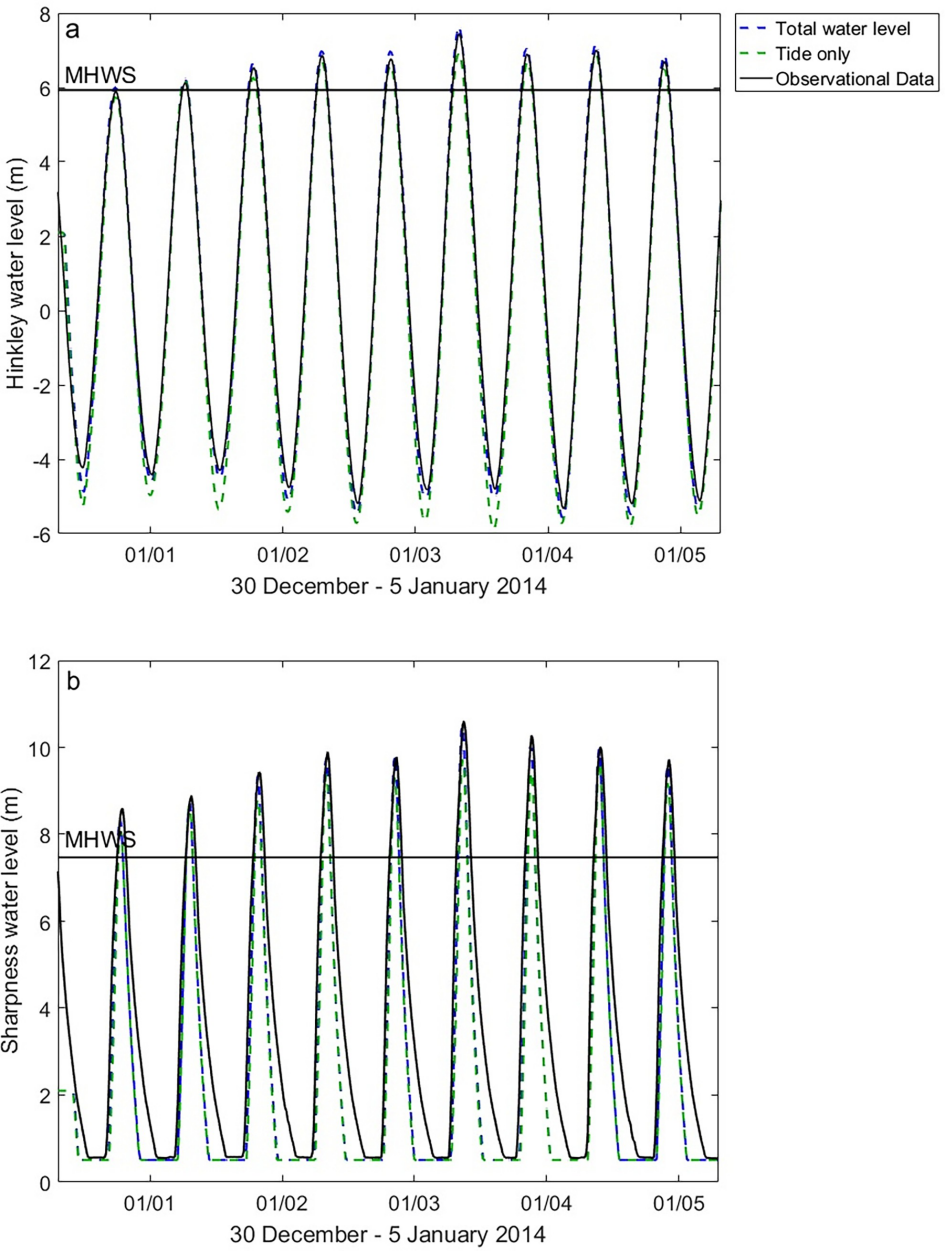
Model output validation for realistic timing of total water level and tide only model runs compared to observational data at a) Hinkley Point and b) Sharpness tide gauge, Severn Estuary, southwest England.

Delft3D-FLOW, a hydrodynamic, numerical model [41], is used in this study to simulate barotropic tide-surge-river propagation across a two-dimensional horizontal, curvilinear grid, in the Severn Estuary (Fig 1). Gridded bathymetry data at 50 m resolution [42] were interpolated onto the model grid, and a uniform Manning friction coefficient of 0.025 is applied to the grid. The sensitivity of the model to the Manning friction co-efficient was tested by running a 99th percentile water level event (3 January 2014), at varying friction values (0.015, 0.02, 0.025, 0.03, 0.035, 0.04) over a 5 day period. These 6 values were selected based on previous works studying tide-surge propagation in coastal and estuarine systems [43–46]. The value of 0.025 for the Manning coefficient was selected because it gave the best agreement with tide gauge observations (Fig 2A and 2B). The final mesh was chosen following an iterative process of refining the grid to resolve the channel-bank system and the tidal propagation up the estuary. A domain of this size will experience limited internal surge generation due to local meteorological forcing [47] so the extent of the domain was located where observations were available to provide an external tide-surge forcing. The model domain has an open boundary to the west, from Rhosilli, Gower Peninsula to Woolacombe, Devon which is forced using 15 minute tide gauge water level data from Ilfracombe and The Mumbles. A river boundary at Gloucester to the east is forced by 15 minute river gauge water level data from Sandhurst. Boundary forcing excludes meteorological or wave forcing, to allow tide and surge propagation from the open boundary to be assessed up-estuary with consideration for the local interaction.

### 2.2 Long-term tide gauge records

The 15-minute frequency, long-term tide gauge records, collected by the UK Tidal Network (https://www.bodc.ac.uk/data/online_delivery/ntslf/), from Ilfracombe and The Mumbles are used to force the water levels in a series of model setups. Four extreme water level events in the tide gauge record exceeding the 99th, 95th and 90th percentile water level values are identified. The storm tide peak of each extreme water level event is isolated, and the water level time series extracted from the record 3 days prior to and 2 days after the storm tide peak.

### 2.3 Tested tide-surge configurations

The surge component provided within the tide gauge record is separated from the 5-day total water level time series (observed total water level–predicted tide). The surge component is separated from the 5-day total water level time series (observed total water level–predicted tide). The predicted, harmonic tidal signal is based on 114 constituents [48] and is removed from observed total water level at tide gauge locations to estimate the residual and ensure any tide-surge interaction remains within this residual surge component [49]. A Chebyshev type II, low-pass filter is applied to the residual surge component to remove all energy at tidal frequencies, using a stop-band of 26-h and a pass-band of 30-h (cf.[50]). The method separates out the time-varying meteorological residual and the tide-surge interactions, which will have been removed by the filter since it is frequency-dependent [50], to leave only the long period surge component. Atmospheric forcing is not included to restrict the sensitivity analysis to tide-surge propagation, without the complication of a locally generated surge contribution.

The filtered surge component is recombined with the predicted tide in a range of time-shifted configurations. The peak of the filtered surge changes relative to the peak of tidal high water to investigate the influence of the timing of the peak of the surge on tide-surge propagation, total water level and surge elevation. The first time series represents the realistic timing of the peak of the surge relative to tidal high water for each of the 4 extreme water level events. A ‘tide-only model’ run is completed to provide the baseline water level which other model runs can be compared to. An additional 13 model time series are created in the time-shift analysis, so the peak of the filtered surge occurs 6 hours before tidal high water and advances incrementally to tidal high water and then continue to 6 hours after, to cover a 12-hour tidal cycle.

### 2.4 Model validation

Model results from the 99^th^ percentile water level event, 3 January 2014, are isolated from the model outputs and standard protocol is followed [51–53] to validate these outputs at the coast with observed data from tide gauges at Hinkley Point, Newport, Portbury, Oldbury and Sharpness. Model outputs for the realistic timing of total water level model run and a tide only run, which provides a baseline, are compared to observation data from the UK Tidal Network, Environment Agency and Magnox. These tide gauge locations are spaced throughout the estuary, and data are freely available to download from the British Oceanography Data Centre, with uninterrupted observational records available for the extreme water level events selected. Widely used error metrics (R^2^ [54], NRMSE [51,55], Bias of the maximum value [55,56]) are calculated at tide gauge locations up-estuary. These metrics confirm that the model can reproduce observational tide gauge data without the inclusion of meteorological forcing and waves and can be used to assess the error introduced by this methodology.

Fig 2A illustrates validation model runs and observational tide gauge data from Hinkley Point, on the south shoreline of the outer estuary. There is good graphical and statistical agreement between the model output (dashed line) and observational tide gauge data (solid line). Tidal phase is successfully reproduced by the model. The total water level model run is able to reproduce the tide gauge data at Hinkley Point well, with an R^2^ value of 0.996 and NRMSE is 1.59% of observed tidal range. It The water levels predicted by the model are overestimated by 15–20 cm for the total water level run on the largest tide of 3^rd^ January 2014. However with a tidal range of 12.29 m, this over-estimate represents just 1.5% of the overall tidal range. The tide only model run does not resolve the high water peaks, indicating the importance of the inclusion of a meteorological surge component in total water level estimations.

Fig 2B shows model validation at Sharpness river gauge further up-estuary. There is a notable asymmetry in the tidal phase due to shallow water impacts, which are accurately simulated by the model. The total water level model run is able to reproduce the tide gauge data at Sharpness well, with an R^2^ value of 0.985 and NRMSE is 1.63% of observed tidal range. The results of the validation indicate that the model output is in good agreement with the observations, for the size and resolution of this model domain (see Fig 1), and is able to reproduce extreme water levels without the inclusion of meteorological forcing and waves.

The following modeled variables have been analyzed:

- Surge, which is modeled total water level–modeled tide;
- Maximum surge elevation;
- Tidal range, which is modeled mean high water–mean low water;
- Surge range, which is maximum modeled surge elevation–minimum modeled surge elevation;
- Variability in surge elevation, bound by the maximum and minimum surge elevation;
- Variability in skew surge elevation, bound by the maximum and minimum skew surge elevation.

## 3. Results

The model confirms that there is uncertainty in the predicted surge levels at tide gauge locations in the Severn Estuary. First, the surge component is isolated at the tidal gauge locations, and is then analyzed relative to the tide. Second, the tidal range, the surge range, and the variability in the surge and in skew surge elevations for time shifts are analyzed along the thalweg of the estuary, for each of the four extreme water level events that occurred in the period being analyzed.

### 3.1 Analysis of surge elevation from 1^st^– 5^th^ January 2014

**Fig 3:**
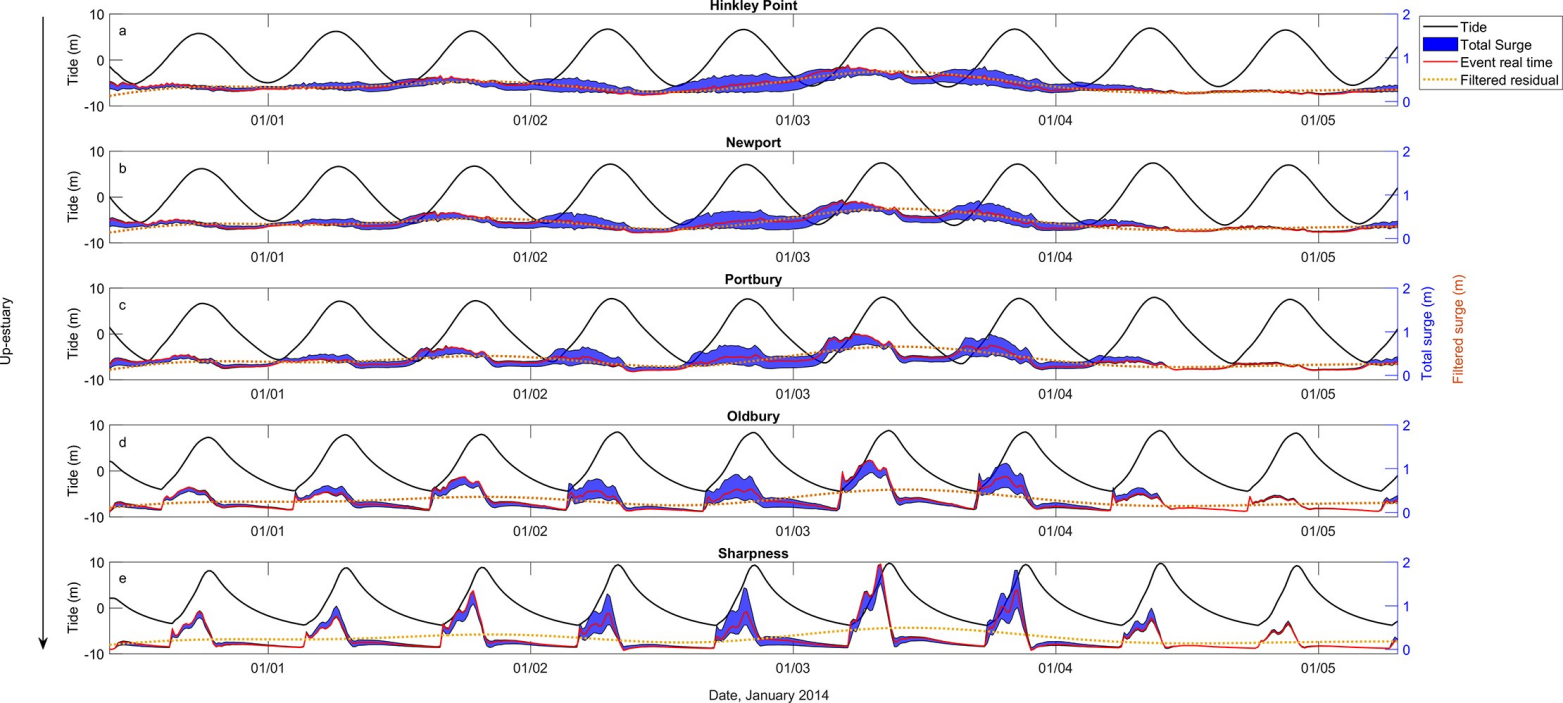
Modeled tidal time series (black); modeled surge elevation for the realistic surge timing (red line); range of surge elevations for time shifted configurations shaded (blue band); observed filtered surge (orange line) at a) Hinkley; b) Newport; c) Portbury; d) Oldbury; e) Sharpness for the 3^rd^ January 2014 event.

The surge is presented over the 5-day model simulation for 99^th^ percentile water level event, 3 January 2014, at tide-gauge locations up-estuary (Fig 3). The 5-day, shaded time series (seen in blue in Fig 3) captures the full range of potential surge elevations as the filtered surge is moved in time around tidal high water at the open boundary.

Fig 3 shows that the maximum of the surge elevation occurs on the 3rd January 2014, increases towards the river side of the estuary. The maximum elevation of the surge is 0.84 m at Hinkley Point at 05:00. The maximum elevation of the surge increases to 0.88 m at Newport and occurs at the earlier time of 04:30. The maximum elevation of the surge increases further to 0.99 m at Portbury at 05:30 and 1.2 m at 07:00 at Oldbury. The surge reaches maximum elevation, 1.96 m, at Sharpness at 08:00.

Fig 3 shows maximum surge elevation consistently occurs during flood tide, regardless of the phase shift of the filtered surge at the boundary around tidal high water. Maximum surge elevation at Hinkley Point occurs at 05:00 time on 3 January 2014, which is 2.5 hours before modeled tidal high water. Maximum surge at Sharpness occurs closer to high water at 08:00 time on 3 January 2014, which is 45 minutes before modeled tidal high water.

The shape of the surge curve exhibits noticeable changes as it propagates up-estuary. At Hinkley Point and Newport the surge residual displays long duration, low magnitude elevations. At Portbury, Oldbury and Sharpness the surge curve has short duration, high magnitude elevations and exhibits an M2 tidal signal. This may be caused by the funneling effect, due to channel convergence [57], which amplifies surges from the deeper part of the outer estuary, through the increasingly narrow, shallow channel towards Portbury [38]. The funneling effect, which amplifies the surge up-estuary, is thus likely to be the driver for an increased positive surge contribution to the total water level up-estuary.

The red line on Fig 3 highlights modeled surge elevation for the observed (realistic) timing of the surge on 3 January 2014. The peak of the surge occurs 3 hours after tidal high water at the open boundary, and shows variability in its positioning within the blue band. This demonstrates that each time shift does not cause a consistent surge response over the duration of the modeled event. The dotted orange line on Fig 3 shows the filtered modeled residual (modeled total water level–modeled tide). The filtered residual shows a reduction in amplitude up-estuary. This may be due to the influence of quadratic bottom friction as the channel becomes shallower [49]. Frictional influences cause loss of energy in the movement of water to dampen the surges’ amplitude as it propagates up-estuary [29]. The reduction in filtered surge indicates the increase in total surge, and is likely to be a consequence of increasing locally generated tide-surge interaction up-estuary to a point where this interaction dominates the shape of the surge curve.

Table 1 shows the contribution of the surge to total water level (prescribed here as a mean sea level, astronomical tidal curve, representative surge curve and freshwater input) (1) when the surge reaches a maximum, (2) at the time of tidal low water and (3) at the time high water at each location.

**Table 1:** Contribution of surge to total water level at the time of maximum surge (total water level–predicted tidal level), tidal low water and tidal high water.

	**Contribution of surge to total water level at changing times (%)**		
	Maximum surge	Tidal low water	Tidal high water
**Hinkley Point**	11.34	88.57	5.14
**Newport**	12.92	92.03	5.02
**Portbury**	17.49	94.39	4.57
**Oldbury**	15.50	40.3	7.09
**Sharpness**	12.14	27.91	5.37

The surge contributes 11.34% to total water level at Hinkley Point and 12.92% at Newport when it reaches a maximum elevation on 3 January 2014. The contribution of the surge to total water level reaches a peak of 17.49% at Portbury, and then decreases up-estuary to 12.14% at Sharpness. The contribution of the surge to total water level at the time of the surge maxima declines up-estuary despite generating the greatest overall surge elevation. The peak of the surge at locations up-estuary occurs closer to tidal high water than at the down-estuary locations. This larger tidal elevation acts to mask the contribution of the surge.

The surge contributes a greater proportion to total water level at tidal low water. The surge contributes a maximum of 94.39% to total water level at Portbury at the time of tidal low water. The contribution of the surge to total water declines further up-estuary at Oldbury and Sharpness at tidal low water. The contribution of the surge to total water level at the time of tidal high water is small in comparison to tidal low water, and reaches a maximum at Oldbury. The variability in the high and low water contributions is partly due to the phase of the surge peak relative to these times, which is why the skew surge parameter becomes important. These results show the largest contributions occur approximately where the tidal range is also at its largest [58] due to the funneling influence of the estuary.

### 3.2 Surge elevation along thalweg

Fig 3 shows that the maximum surge elevation increases and occurs closer to tidal high water as it propagates up-estuary, and there is greater influence of tidal harmonics on the surge up-estuary. A change in the surge as it propagates through the estuary therefore influences flood hazard up-estuary. Tidal range, surge range, and variability in the surge and skew surge elevations along the entire estuary, and through the deepest channel (thalweg) for four extreme water level events are presented in Fig 3.

**Fig 4:**
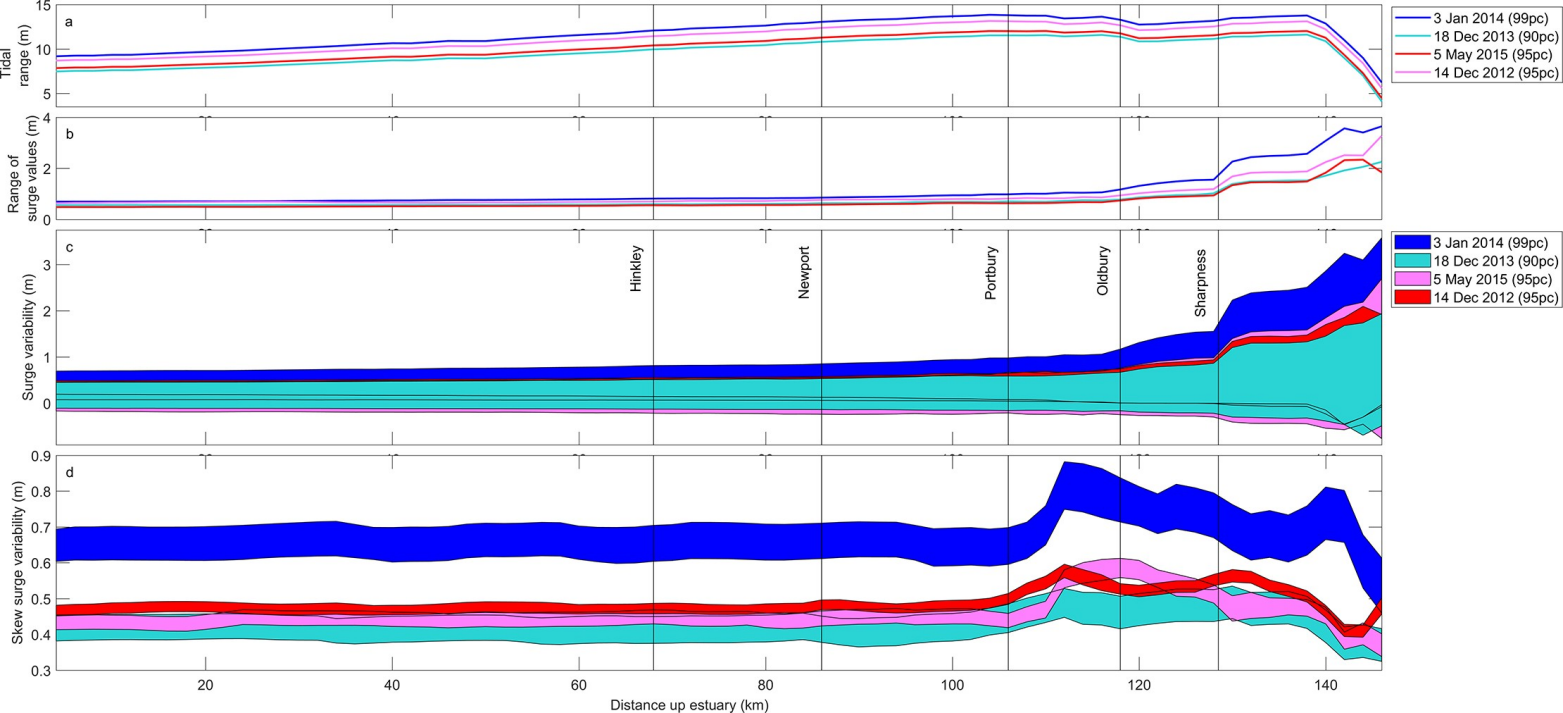
a) Tidal range; b) Surge elevation range for observed event timing; c) Variability in surge values; d) Variability in skew surge values for time shift configurations along thalweg of the Severn Estuary.

Fig 4 shows that the tidal range (Fig 4A) increases linearly in the lower estuary, from the open model (sea) boundary to Portbury. The tide is funneled through the estuary to a maximum range of 13.85 m at Portbury (cf. [38]). The tidal range remains approximately constant from Portbury to a point beyond Sharpness, some 140km from the open end of the model, potentially due to frictional influences. The range in surge elevations (maximum surge elevation–minimum surge elevation) (Fig 4B) and variability in elevation due to the timing of the peak of the surge relative to tidal high water (Fig 4C) remain constant from the model boundary to Newport. Both display an exponential increase from Portbury beyond Sharpness. The rate of increase is seen to be greater for a more severe storm. This indicates the system becomes more sensitive to the surge up-estuary. It is noticeable that the 99^th^ percentile water level event (3^rd^ January 2014) produces the greatest variability in surge elevations beyond Portbury, with a maximum elevation of 3.58 m beyond Sharpness.

The variability in skew surge elevations due to the time-shifted configurations (Fig 4D) is constant down-estuary for all extreme water level events, similar to the trend seen in the range and variability of surge elevations and tidal range. Skew surge values increase more rapidly than the surge, beyond Portbury, due to the tidal range starting to become damped by friction and the surge starting to increase due to enhanced interaction. The skew surge values decay up-estuary where there is a dominant asymmetrical signal in the tide and surge. This trend is similar for all extreme water level events, for all time shifts. The 99th percentile water level event (3 January 2014) and 90th percentile water level event (18 December 2013) show greatest sensitivity to timing, with greater variability in skew surge values. The 99th percentile water level event (3 January 2014) consistently displays the greatest skew surge values along the channel of the estuary, reaching a maximum of 0.88 m at 112 km up-estuary.

Portbury presents a tipping point in the balance between funneling and frictional influences, and a change in the dominant contribution to total water level. The funneling effect acts to increase tidal range up to Portbury, the tide is also the dominant influence on total water level down-estuary. Frictional influences dominate beyond Portbury to dampen the tidal range, which is most noticeable beyond Sharpness. It is suggested that while frictional influence dampens the tidal range it also acts on both tide and surge to enhance asymmetry in the time series, causing the surge range to increase up-estuary in response to tide-surge interaction (Fig 3). The relative contribution of the surge to the total water level thus increases past this point.

## 4. Discussion

Variability in the total surge and tide-surge interaction can cause large uncertainties in flood-hazard assessment, which can be a significant concern for coastal asset managers who rely on accurate predictions of total water level for storm hazard mitigation. Here, the barotropic tide-surge-river propagation and interaction across a hyper-tidal estuary has been investigated using the Severn Estuary as test case, and the numerical model Delft3D-FLOW.

The model highlights the influence of the timing of the peak of the surge relative to tidal high water on the surge elevation for 4 historical events in a hyper-tidal estuary, where small changes in surge timing and magnitude can have significant implications for total water levels [4]. A shift in the timing of the peak of a storm surge nearer to the time of tidal high water can elevate water levels and increase the risk of overwashing or overtopping of coastal defenses [14,59]. The surge contains a meteorological component and a tide-surge interaction component, and is calculated by removing the tide from the total modeled water level. Surge elevation, surge range, variability in surge and skew surge elevation along the channel, and at gauge stations throughout the estuary are presented.

The model confirms the magnitude, duration and shape of the surge (including filtered external surge and local tide-surge interaction) changes up-estuary. On the 99th percentile water level event, the surge component amplifies up-estuary as it becomes increasingly asymmetrical and peak water levels occur when the surge is closer to high water. The model clearly illustrates how estuary morphology amplifies tidal wave and surge propagation up-estuary due to topographic features or changing bathymetry [60,61].The model confirms the importance of shallow water interactions in amplifying tides and storm surges through an increasingly shallow, narrow estuary, which can act to elevate flood hazard [58]. The Thames Estuary shows similar behavior as shallow water interactions have a time-displacement effect on tidal propagation [62]. A positive surge can increase phase speed of tidal propagation to alter the timing of tidal high water which is critical for flood hazard during the time of a spring tide and large, positive surge [20]. Analysis of residuals from tide gauges in the North Sea show that advancement of the surge at tidal high water is of the greatest practical significance for operational forecasts of sea-level [4]. It is of critical importance for surge predictions to consider more than a linear super-position of predicted tide and forecast meteorological surge in shallow water regions, as tide-surge interactions amplify surge elevation and alter the timing of tidal high water [23].

In shallow water areas dynamic processes can cause the tidal and surge components to interact, and become increasingly distorted and asymmetrical [63,64]. This is a common phenomenon in the Meghna Delta, Bangladesh, where tidal range exceeds 4.5 m and bottom friction effects influence the timing and magnitude of high water [65].The rate of rise of the water level is more rapid than the rate of fall due to decreasing depth of the channel, giving rise to asymmetrical surges [66]. Surges are amplified up-estuary and become more asymmetrical in shape, controlled by channel convergence and contribute more to total water levels, until a tipping point where bottom friction becomes a more important control on surge attenuation [57]. Channel convergence and shallow water effects could modify the shape of the curve prior to tidal high water to alter the duration of high water, and lead to uncertainty in duration and volume of water affecting a region. Modification of the shape of the surge curve up-estuary due to shallow water effects must be captured in surge predictions to avoid incorrect total water level forecasts, which in turn could lead to an increased risk of loss of life and damage to property.

The effect of shallow water on amplifying surge elevation and increasing total water level is well documented in other narrow, hyper- and macro-tidal estuaries worldwide. In the Taiwan Strait, where tidal range exceeds 4 m, nonlinear bottom friction and channel convergence intensifies tide-surge interaction to enhance tidal elevation [67]. The influence of tide-surge interaction on peak water levels also depend on the path of typhoons generating the surge and wind direction [8]. Local conditions e.g. shallow bathymetry [19], storm characteristics [68] and tide-river flow interactions [69] may also contribute to changes in surge elevation in shallow water areas, complicating surge predictions. The model highlights the importance of physical processes on total water levels which occur in coastal zones worldwide, not just in estuaries. Simulations of storm surges induced by Hurricane Rita on the Louisiana-Texas coastline present bottom friction as the most important term for surge amplification in nearshore areas [68]. The importance of considering tide-surge as combined, coupled, physical processes in total water level predictions is shown on the North Queensland coast, where bottom friction can lower total water levels [18]. It is shown that the addition astronomical tide and meteorological storm surge can overestimate total water level, causing errors in early warnings [18].

The maximum surge elevation consistently occurs on the rising tide, regardless of the phase shift of the filtered surge relative to tidal high water. This effect has been noted in other shallow water regions and estuaries. It has been shown in the North Sea that maximum skew surges are more likely to occur 3–5 hours before tidal high water which can amplify surge magnitude as the shallow water wave travels through deeper water [4]. Noticeable surge influence on the rising tide should be incorporated in the surge curve shape used for flood risk analysis and considered as a source of uncertainty in surge forecasts when applied for up-estuary locations.

The results show noticeable changes in the range of surge elevations through the channel, when the realistic timing of the extreme water level event is analyzed. The range of surge elevations across all four extreme water level events is constant down-estuary, increases slowly beyond Newport and continues to increase exponentially beyond Portbury across all four extreme water level events. The greater range of potential surge elevations that could occur up-estuary may be due to the greater contribution from tide-surge interaction to total water level in these shallow regions. In the Bay of Fundy, Canada, mean spring tidal range can exceed 15 m, which makes it the largest in the world [70]. Storm surges gain elevation up-estuary due to near-resonance with North Atlantic tides and strong frictional effects due to shallow water depths [59]. Analysis of sea level observations shows that tide-surge interaction, due to bottom friction, can elevate total water level up to 20 cm in the Northumberland Strait during severe storm events [19,24]. The contribution of tide-surge interactions to total water level is of practical significance in terms of water level forecasts and flood hazard assessment. Uncertainty in a storm tide time series, especially when the peak of the surge occurs close to tidal high water (when exceedance of critical thresholds, e.g. defenses, is likely to occur) could influence overflow volume and defense overtopping [9]. Therefore the shape of surge curves which form basis of flood hazard assessment up-estuary could be wrong for the inner estuary regions if increased variability in maximum surge elevation up-estuary is not accounted for. Incorrect predictions of potential flood hazard will have significant practical implications for communities and critical infrastructure located on low-lying land, with the potential for damage to property and people. At sites of high value (in this case in terms of energy infrastructure) valid modelling tools are required to evaluate coastal resilience. Using a validated model this study quantifies uncertainty surrounding extreme water level forecasts due to tide-surge interaction. Better understanding of this uncertainty informs decisions made by policymakers who set and plan coastal flood response strategies.

Communities and industries developed on low-lying land near tide dominated estuaries (hyper-tidal in this case) require accurate storm surge prediction systems for effective flood hazard mitigation plans and flood warning. Early flood warning in the UK is based on a forecasting system which combines predicted tides and forecasted surges at tide gauge locations in the UK, from the CS3X storm surge model [71]. An error in flood hazard assessment could occur for tide gauge locations where forecasts are made available due to increased variability in maximum surge elevations and greater magnitude, shorter duration of surge curves, most notably in locations where flood hazard assessments are based on the surge forecast for down-estuary locations. A total water level prediction for a location up-estuary, which is estimated using down-estuary tide gauge data, could lead to total water level being under-predicted. An under-prediction of total water level will have consequence for the duration flood water may be able to inundate a site [9]. Very often the surge forecast is used to forecast a skew surge value relative to the predicted tide: it is this parameter that provides an indicator for the likelihood of flooding, although it does not indicate the timing or the duration.

The UK Met Office and National Oceanography Centre provide sea-level forecasts, including skew surge predictions, for tide-gauge locations within the Severn Estuary using tidal prediction and surge forecasts [72]. The results (Fig 4D) show that beyond Portbury there is greater variability in maximum skew surge elevations, indicating sensitivity to the timing of the surge relative to tidal high water. The up-estuary response of skew surge does not follow that of the surge: skew surge values decline beyond Oldbury as tide and surge become increasingly asymmetrical. Therefore surge elevation is low at the time of tidal high water and large when the rising tide is close mean water level. The consequence is a low surge contribution during elevated tidal levels and thus a reduced skew surge up-estuary.

## 5. Conclusion

Variability in the storm surge component of total water level needs to be captured accurately to reduce uncertainty in site specific hazard assessments. This is especially the case in hyper-tidal estuaries, where the tidal range may exceed 6m, and the surges can be amplified towards the head of the estuary, increasing flood risk in that region.

This research has shown that maximum surge elevations increase up-estuary, with surge curves displaying greater magnitude and shorter duration. A total water level prediction for a location up-estuary, which is estimated using down-estuary tide gauge data, could lead to total water level being under-predicted, and will have consequence for the duration that flood water may be able to overwash coastal defenses. Local forecasting systems, which rely on accurate estimations of storm surge, should consider changes in surge elevation and shape with distance up-estuary from nearby tide gauge sites.
